# Ecological Factors Associated with European Bat *Lyssavirus* Seroprevalence in Spanish Bats

**DOI:** 10.1371/journal.pone.0064467

**Published:** 2013-05-20

**Authors:** Jordi Serra-Cobo, Marc López-Roig, Magdalena Seguí, Luisa Pilar Sánchez, Jacint Nadal, Miquel Borrás, Rachel Lavenir, Hervé Bourhy

**Affiliations:** 1 Centre de Recerca en Infeccions Víriques, Illes Balears (CRIVIB), Fundació d’Investigació Sanitària de les Illes Balears, Conselleria de Salut i Consum, Govern de les Illes Balears, Palma de Mallorca, Spain; 2 IRBIO and Departement de Biologia Animal, Facultat de Biologia, Universitat de Barcelona, Barcelona, Spain; 3 Centro Nacional de Epidemiología, Instituto de Salud Carlos III, CIBERESP, Madrid, Spain; 4 Unitat de Toxicologia Experimental i Ecotoxicologia, Parc Científic de Barcelona, Barcelona, Spain; 5 Institut Pasteur, Unité Dynamique des *Lyssavirus* et Adaptation à l’Hôte, Paris, France; CSIRO, Australia

## Abstract

Bats have been proposed as major reservoirs for diverse emerging infectious viral diseases, with rabies being the best known in Europe. However, studies exploring the ecological interaction between lyssaviruses and their natural hosts are scarce. This study completes our active surveillance work on Spanish bat colonies that began in 1992. Herein, we analyzed ecological factors that might affect the infection dynamics observed in those colonies. Between 2001 and 2011, we collected and tested 2,393 blood samples and 45 dead bats from 25 localities and 20 bat species. The results for dead confirmed the presence of EBLV-1 RNA in six species analyzed (for the first time in *Myotis capaccinii*). Samples positive for European bat lyssavirus-1 (EBLV-1)–neutralizing antibodies were detected in 68% of the localities sampled and in 13 bat species, seven of which were found for the first time (even in *Myotis daubentonii*, a species to date always linked to EBLV-2). EBLV-1 seroprevalence (20.7%) ranged between 11.1 and 40.2% among bat species and seasonal variation was observed, with significantly higher antibody prevalence in summer (July). EBLV-1 seroprevalence was significantly associated with colony size and species richness. Higher seroprevalence percentages were found in large multispecific colonies, suggesting that intra- and interspecific contacts are major risk factors for EBLV-1 transmission in bat colonies. Although bat-roosting behavior strongly determines EBLV-1 variability, we also found some evidence that bat phylogeny might be involved in bat-species seroprevalence. The results of this study highlight the importance of life history and roost ecology in understanding EBLV-1–prevalence patterns in bat colonies and also provide useful information for public health officials.

## Introduction

High species diversity (about 1,150 in the world), worldwide distribution, high mobility and the fact that they represent a continuing source of emerging infections for humans make bats one of the most epidemiologically relevant groups of mammals to study disease ecology. Indeed, bats were shown to be involved in several emergent viral diseases (Coronaviruses, Flaviviruses, Astroviruses, and Adenoviruses etc.), with rabies being one of them [1],[2]. Numerous bat species have been found to be infected by lyssaviruses [3] and bats serve as the reservoirs of 10 of the 11 *Lyssavirus* species described, suggesting that the lyssaviruses originated in these mammals and progressively diverged from a common ancestor [4],[5]. Two new recently described tentative of the three novel *Lyssavirus* species further enlarged the genetic diversity of lyssaviruses found in bats [6]–[8]. In Europe, two *Lyssavirus* species, European bat *Lyssavirus* Types 1 and 2 (EBLV-1 and EBLV-2, respectively), and one tentative species, Bokeloh bat lyssavirus, circulate among several bat species [7]. EBLV-1 is widely distributed throughout Europe and two variants have distinct distributions and evolutionary histories: one is EBLV-1a, which has an east–west distribution from Russia to France, with very little genetic variation; and the other is EBLV-1b, which exhibits a south–north distribution and far more genetic diversity [9].

The first *Lyssavirus* infections in European bats were diagnosed in 1954 in Serbia–Montenegro [10] and Germany [11]. The number of positive cases increased considerably from 1985, when several European countries began routine passive surveillance. From 1977 to 2012, 1033 bats were found to be infected with lyssaviruses in Europe (http://www.who-rabies-bulletin.org). The substantial number of positive bats diagnosed, the number of European countries affected and, above all, the finding that EBLV-1 and EBLV-2 can cross the species barrier to infect other domestic and wild non-flying mammals and humans raised public health issues related to these and other viruses [12],[13].

Most EBLV-1–positive European bats were identified during passive surveillance and diagnosed in the Serotine bat (*Eptesicus serotinus*) [14]. Few data are available on the infection incidence in other European bat species. However, active surveillance indicated that several other bat species has serological evidence of previous infection. The role of these species in EBLV-1 epidemiology, particularly *Lyssavirus* cross-species-infection dynamics, remains unknown. Given the fact that *E. serotinus* is a non-migratory bat [15], it is possible that migratory species may have a more important role in the dispersion [16],[17] and distribution of the different EBLV-1 genetic variants. With the aim of understanding more about the role of the different bat species in EBLV-1 dynamics and identifying ecological factors that might favor EBLV-1 transmission and, consequently, serological responses to infection in bat colonies, active surveillance of bat colonies in Spain was implemented in 1992. In this study, we analyzed ecological and epidemiological factors that might be associated with the infection dynamics observed in colonies where we previously detected EBLV-1 infection [17]–[19], and completed with data collected during 2001–2010.

## Materials and Methods

### Ethics Statement

All animals were handled in strict accordance with good animal practices, as defined by current European legislation. Bat capture and blood-sampling were authorized by permit from the Spanish Regional Committee for Scientific Capture.

### Sample Collection

**Figure 1:**
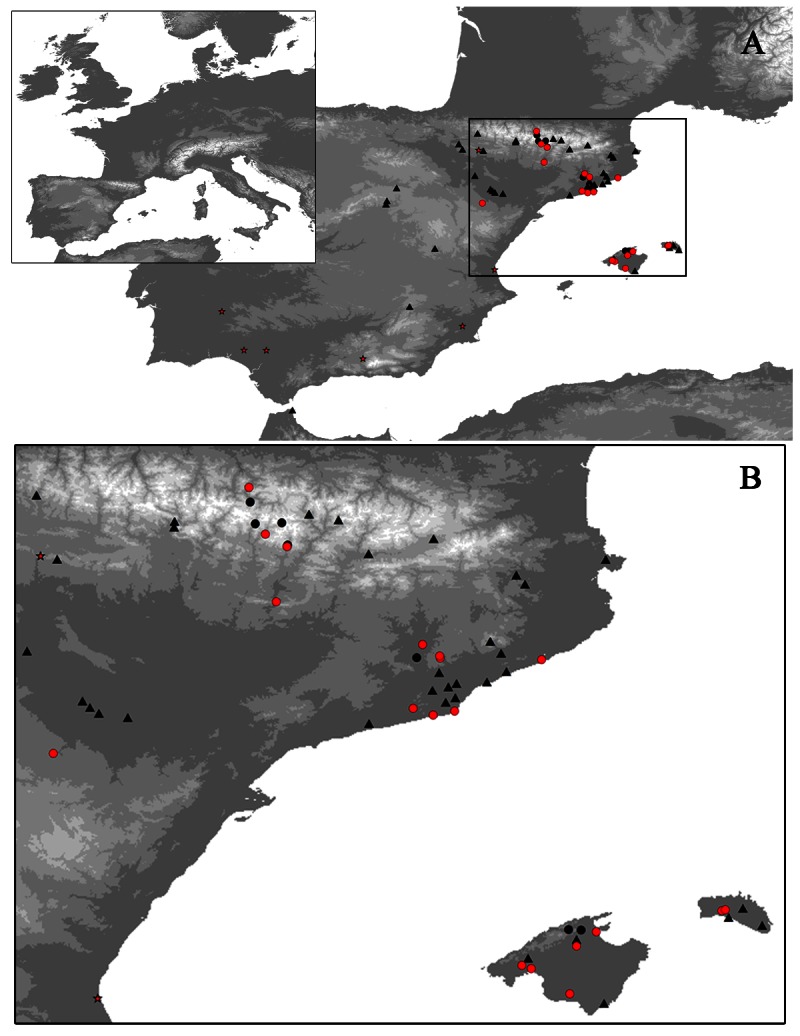
Map of the Iberian Peninsula showing the localities sampled. (A) Bat-sampling locations in Spain, 1992–2010. (B) Expanded area showing the localities sampled for this study. Red circles indicate localities where seropositive bats or individuals with EBLV-1 RNA were found, and black circles indicate seronegative localities sampled. Black triangles indicate previously sampled localities [17]. Stars indicate bibliographic cases of EBLV-1 infection [17],[31]–[36].

From 2001 through 2010, bats were collected from 25 localities in three autonomous Regions: Aragon, Balearic Islands and Catalonia (Figure 1). Localities were selected on the basis of bat- behavior criteria: synanthropic (urban areas), migratory and gregarious species. Bat colonies were sampled throughout the year, avoiding hibernation (from mid-December to the end of February) and the birthing periods (from mid-June to mid-July).

Insectivorous bats were captured inside the roosts with long-handled butterfly nets during the day or with mist nets at sunset, when they emerged to forage. The latter nets were used only when access to the roost interior was not possible. Thick leather gloves were worn when bats were handled and transferred into individual cotton pouches for transportation and processing.

All bats were identified to species, based on the identification key to the bats of Europe [20]. Individuals were sexed and aged as juveniles or adults based on the degree of epiphyseal fusion [21]. Reproductive status of adult females was classified as pregnant or lactating, based on palpation of the abdomen and nipple condition [22]. For future long-term studies on population dynamics only in some localities, bats were banded with a uniquely coded alloy ring (Porzana Limited, East Sussex, UK) on the forearm.

### Bat Sampling

Bats were arbitrarily captured and blood was drawn for analyses. Bats identified as juveniles were not analyzed in this study. However, some individuals, whose age category was difficult to determine precisely according to the criterion applied, were included in the statistical analysis. Blood samples (0.1–0.5 mL, depending on the bat's size) were obtained by a small puncture made in median artery. Pressure with a sterile towel was applied to the wound until the bleeding stopped and a sterile absorbent hemostatic sponge impregnated with gelatin was place on the site to prevent bleeding and facilitate healing, and the bat was released. The bats were offered 10% glucose–water orally to prevent dehydration and provide rapidly assimilated compounds for energy. Vials containing blood were stored at 4°C for a few hours. Samples were centrifuged for 20 minutes at 12,000 rpm, and the serum was extracted with a micropipette. Serum samples and clot pellets were frozen at −20°C and –80°C respectively, before analysis. The 45 carcasses analyzed during the study were dead bats found during fieldwork or those that died during handling. The bats were not further discriminated into subgroups based upon whether they were found dead or died during processing, with the latter deaths probably being attributable to cardiomyopathy or other stress; none exhibited any symptoms associated with rabies. Independently of blood samples, brain, pharynx–esophagus, larynx, lung, heart and tongue samples were collected aseptically from dead bats in the laboratory and stored at –80°C.

### Detection of EBLV-1 Neutralizing Antibodies

The technique used to detect EBLV-1 neutralizing antibodies is an adaptation of the Rapid Fluorescent Focus Inhibition Test (RFFIT) [17],[23]. A constant dose of a titrated (calibrated to give 80% fluorescent foci (infected cells)), cell-culture–adapted, EBLV-1 challenge virus (8918 FRA) was incubated with 3-fold dilutions of the sera to be titered. After incubation of the serum–virus mixtures, a suspension of BSR cells was added. Twenty-four hours later, the cell monolayer was acetone-fixed and labeled with a fluoresceinated anti-nucleocapsid antibody (BioRad, Marnes-la-Coquette, France) to detect the presence of non-neutralized virus (fluorescent foci). The optimal challenge dose (the dilution giving 80% infected cells for each virus production) is calculated. Further, titers are expressed as the arithmetic means of two independent repetitions. Samples were considered positive when the number of fluorescent foci was reduced by 50% at the 1∶27 dilution (starting dilution). This cut-off value is similar to that applied in other studies [17],[19],[24].

### Detection of EBLV-1 RNA

Total RNA was extracted from individual blood clots and organs, and tested by nested real-time polymerase chain reaction (nRT-PCR) [17].

RNA was extracted in a P3 laboratory. Then, template preparation, RT-PCR mix preparation and DNA addition to the mix were done using aerosol-resistant tips in two distinct rooms. In all these procedures, negative controls were performed individually for each step (extraction, reverse transcription, first and second PCR) and were negative. In addition, RNA extracted omitting reverse transcriptase was also subjected to nRT-PCR to serve as controls. A 394-bp amplicon of the nucleoprotein gene was obtained with primers N41 and N60. The second PCR amplicon (N62–N63) was 161-bp. The Sanger method was used to sequence the PCR products, which were analyzed with Sequencher 10.1 software. The 161-bp sequences obtained by the second PCR with the N62–N63 primer set were compared. They were blasted against Genbank.

### Statistical Analyses

In an attempt to identify ecological factors associated with EBLV-1–antibodies prevalence, we analyzed the probability of being ELBV-1–seropositive as a function of five explanatory variables: taxon, month, sex, colony size and species richness. In this statistical analyses we only consider the months included, from April to October, period during which the bats are more active and occur higher infection rates [25],[26]. The taxon variable included four families of bats sampled in Europe (*Rhinolophidae*, *Vespertilionidae*, *Miniopteridae* and *Molossidae*) [27].

The colony size was estimated at each sampling time and for each species found in the roost from direct census conducted inside the refuge or when bats had left the roost to forage at night. Because accurate colony-size estimates were only available for some localities, we categorized the colony-size variable as small, medium or large. We considered colonies not exceeding 100 individuals small, those harboring 100–500 individuals medium and those home to ≥ 500 individuals large. For each sampling time, we also calculated the number of species (species-richness variable) present in the refuge that form clusters, independently of whether the species were sheltering separately or in proximity to other species. Solitary individuals, mostly of the *Rhinolophus* genus, were not considered in species richness. Sibling species, such as *Myotis myotis* and *Myotis blythii*, and *Pipistrellus pipistrellus* and *Pipistrellus pygmaeus*, were assimilated to form two groups due to the difficulty of identifying them when they were not captured. We also categorized the species richness as 1, 2 or ≥3 species.

Prior to the analysis, we checked for potential collinearity by using the variance-inflation factors (VIF) from a standard linear model, excluding the random effect to assess the absence of multicollinearity among the explanatory variables selected. Because all VIF values were <2, we considered that collinearity was not a serious issue for this data set [28].

We used a generalized linear-mixed model and assumed a binomial distribution to investigate the relationships among EBLV-1 seroprevalence and the five explanatory variables. The 74 distinct sampling times were distributed over 16 localities and 10 years (Table S1 in File S1). We did not take in consideration of those localities with the small numbers of captured bats (fewer than 7 individuals). We excluded also five individuals of undetermined sex. For this analysis, we used 2,144 sera from 12 bat species. To control for variability due to several sampling times among years and localities, the corresponding variables were included as random effects in the models. We also excluded the few bats captured more than once within the same month.

We used an information–theoretic procedure and the Akaike information criterion corrected for small sample sizes (AICc) to compare models [29]. We generated a set of different models that consisted of all combinations of the five explanatory variables. All the models considered for the analysis included the fixed additive effects of the five explanatory variables and the random effects of year and site. For each model *i*, we computed the Akaike weight (w_i_), which can be interpreted as the likelihood that model *i* is the best model within the set in terms of trade-offs between data fit and parsimony. For each independent explanatory variables, we calculated the sum of Akaike weights (∑w_i_), computed for these 16 models in the set including that variable [30]. Finally, we created other models from the best model selected in the previous set that incorporated biological interactions among the explanatory variables and the random-effect terms. Odds ratios and their 95% confidence intervals were computed for the explanatory variables of the resulting model.

All analyses were conducted using the R package version 2.14.2 [31]. Models were run with the ‘glmer’ function in ‘lme4’, using the Laplace approximation of the maximum-likelihood and a logit link function. VIF were calculated using the function “vif” from the R package ‘car’ [28], and likelihood ratio tests between models were calculated using the R-function ‘anova’. McNemar's test was calculated using the “mcnemar.test” function from the R package to investigate significant differences between ELBV-1–seropositivity and taxon (different families).

## Results

### Serological Analysis

Among the 2,393 sera obtained, 495 (20.7%) were positive for EBLV-1–neutralizing antibodies. Among the 25 different Spanish localities, 17 (68%) harbored positive bats (one in Aragon, seven in the Balearic Islands and nine in Catalonia) (Figure 1) [17],[32]–[37]. Fifteen of the 25 localities were sampled for the first time. Highly variable EBLV-1 seroprevalences were observed (3–37%) among localities. EBLV-1–neutralizing antibodies were detected in 13 (65%) of the 20 species analyzed and showed broad variations among bat species (11.1–40.2%) (Table 1), representing the first time that EBLV-1–neutralizing antibodies were detected in *P. pipistrellus*, *Pipistrellus kuhlii*, *Hypsugo savii*, *Myotis daubentonii*, *Myotis escalerai*, *Myotis capaccinii* and *Plecotus austriacus*.

**Table 1:** Serological results of EBLV-1 neutralizing antibodies analyses in Spanish bats (2001 – 2010).

Species	Females		Males		Total	
	No. of samples collected	No. (%) of positive samples	No. of samples collected	No. (%) of positive samples	No. of samples collected	No. (%) of positive samples
*Eptesicus serotinus*	109	19 (17.4)	7	0 (0.0)	116	19 (16.4)
*Hypsugo savii*	6	0 (0.0)	16	5 (31.2)	22	5 (22.7)
*Myotis blythii*	10	2 (20.0)	56	12 (21.4)	66	14 (21.2)
*Myotis capaccinii*	97	13 (13.4)	48	6 (12.5)	145	19 (13.1)
*Myotis daubentonii*	2	0 (0.0)	32	4 (12.5)	34	4 (11.8)
*Myotis emarginatus*	7	0 (0.0)	1	0 (0.0)	8	0 (0.0)
*Myotis escalerai*	34	5 (14.7)	9	1 (11.1)	43	6 (13.9)
*Myotis myotis*	544	225 (41.4)	128	45 (35.1)	672	270 (40.2)
*Nyctalus leisleri*	0	0 (0.0)	3	0 (0.0)	3	0 (0.0)
*Plecotus auritus*	0	0 (0.0)	1	0 (0.0)	1	0 (0.0)
*Plecotus austriacus*	76	12 (15.8)	59	6 (10.2)	135	18 (13)
*Pipistrellus kuhlii*	7	3 (42.8)	9	0 (0.0)	16	3 (18,8)
*Pipistrellus nathusii*	0	0 (0.0)	1	0 (0.0)	1	0 (0.0)
*Pipistrellus pipistrellus*	20	3 (15.0)	25	2 (8.0)	45	5 (11.1)
*Pipistrellus pygmaeus*	2	0 (0.0)	4	0 (0.0)	6	0 (0.0)
*Miniopterus schreibersii*	322	41 (12.7)	219	25 (11.4)	541	66 (12.2)
*Rhinolophus euryale*	0	0 (0.0)	2	0 (0.0)	2	0 (0.0)
*Rhinolophus ferrumequinum*	217	24 (11.1)	79	10 (12.7)	296	34 (11.5)
*Rhinolophus hipposideros*	2	0 (0.0)	1	0 (0.0)	3	0 (0.0)
*Tadarida teniotis*	117	21 (17.9)	121	11 (9.1)	238	32 (13.4)
Total	1,572	368 (23.4)	821	127 (15.5)	2,393	495 (20.7)

### EBLV-1–RNA Analysis

Among the 45 dead bats from seven species analyzed, 12 (27%) were positive by nRT-PCR (Table 2), and EBLV-1 RNA was detected in six species analyzed (*Rhinolophus ferrumequinum*, *M. myotis*, *P. pipistrellus*, *Miniopterus schreibersii*, *Tadarida teniotis* and, for the first time, *M. capaccinii*) (Table 3) [7],[14],[34],[38]–[43].

**Table 2:** EBLV-1 RNA results in Spanish bats (2001 – 2010).

Species	Clots		Organs		Type of organs				
	No. of clots collected	No. (%) of positive clots	No. of bats collected	No. (%) of positive bats	B	Ph-E	L	H	T
*M. capaccinii*	73	0 (0.0)	6	1 (16.7)	+	−	−	−	−
*M. myotis*	557	15 (2.7)	5	1 (20.0)	+	−	−	+	+
*P. austriacus*	101	1 (1.0)	nd	nd					
*P. pipistrellus*	40	1 (2.5)	3	1 (33.3)	−	−	+	−	+
*M. schreibersii* *	376	3 (0.8)	17	3 (17.6)	+	+	−	−	−
*R. ferrumequinum* †	233	18 (7.7)	10	5 (50.0)	+	+	+	+	+
*T. teniotis*	154	5 (3.2)	3	1 (33.3)	−	+	−	−	−
Total	1,823	43 (2.4)	45	12 (26.7)					

B, Brain. E-Ph, Pharynx-Esophagus. L, Lung. H, Heart. T, Tongue. nd, not done.

* details of the individuals positives for each organ: B(1), E-Ph(1), B/E-Ph(1).

† details of the individuals positives for each organ: B(2), E-Ph(1), B/E-Ph(1), B/L/T(1).

**Table 3:** Families and genera of European bat species where *Lyssavirus* infection has been reported (period 1954 – 2011).

Family	*Species*	Lyssavirus RNA	Country	Antibodies	Country
*Miniopteridae*	*Miniopterus schreibersii*	EBLV-1	E	EBLV-1	E,F
	*Miniopterus schreibersii*	WCBLV	R	nd	nd
*Vespertilionidae*	*Eptesicus serotinus*	EBLV-1	CZ,G,DK,E,F,HU,NL,PL,U,A	EBLV-1	E,UK,F
	*Eptesicus isabellinus*	EBLV-1	E	EBLV-1	E
	*Barbastella barbastellus*	EBLV-1	G	EBLV-1	F
	*Myotis capaccinii*	EBLV-1	E	EBLV-1	E
	*Myotis dasycneme*	EBLV-1	NL	nd	nd
	*Myotis dasycneme*	EBLV-2	DK,NL	nd	nd
	*Myotis daubentonii*	EBLV-1	nd	EBLV-1	E
	*Myotis daubentonii*	EBLV-2	CH,UK,F,I	EBLV-2	CH,UK
	*Myotis blythii*	nd	nd	EBLV-1	E,F
	*Myotis myotis*	EBLV-1	G,E	EBLV-1	B,E,F
	*Myotis escalerai*	EBLV-1	E	EBLV-1	E
	*Myotis nattereri*	nd	nd	EBLV-1	B
	*Myotis nattereri*	BBLV	G	nd	nd
	*Nyctalus noctula*	EBLV-1	G	nd	nd
	*Pipistrellus kuhlii*	nd	nd	EBLV-1	E
	*Pipistrellus nathusii*	EBLV-1	G	nd	nd
	*Pipistrellus pipistrellus*	EBLV-1	G,E,F	EBLV-1	E
	*Hypsugo savii*	nd	nd	EBLV-1	E
	*Plecotus austriacus*	EBLV-1	E	EBLV-1	E
	*Plecotus auritus*	EBLV-1	B,G	nd	nd
	*Verpertilio murinus*	EBLV-1	UA	nd	nd
*Rhinolophidae*	*Rhinolophus ferrumequinum*	EBLV-1	E,TR	EBLV-1	E,F
*Molossidae*	*Tadaridas teniotis*	EBLV-1	E	EBLV-1	E

nd, not done. EBLV, *European Bat Lyssavirus*. WCBLV, *West Caucasian Bat Lyssavirus*. BBLV, *Bokeloh bat Lyssavirus*. B, Belgium. CH, Switzerland. CZ, Czech Republic. G, Germany. DK, Denmark. E, Spain. F, France. FI, Finland. HU, Hungary. NL, Nederland. PL, Poland. R, Russia. UA, Ukraine. TR, Turkey. [7],[14],[33]–[44].

Among the 1,823 pelleted blood clots from 18 species analyzed, 43 (2%) contained EBLV-1 RNA (Table 2). Positive clots were found in six (33%) bat species: *R. ferrumequinum*, *P. pipistrellus*, *M. myotis*, *P. austriacus*, *M. schreibersii* and *T. teniotis*. *R. ferrumequinum* (8%) had the highest percentage of individuals with EBLV-1 RNA in clots. EBLV-1 RNA was found in bats captured in seven different locations: one in Aragon, five in the Balearic Islands and one in Catalonia. All positive PCR products were sequenced and the highest Blast score was obtained with previously determined EBLV-1b Spanish isolates (94285 SPA and 9483 SPA) [9],[17] indicating a close relationship between all these isolates and the previously identified EBLV-1b Spanish isolates. A NJ phylogenetic tree was built using a 122-nucleotide long sequence obtained from the blood clots (Figure S1 in File S1).

### Ecological Factors Associated with EBLV-1–Antibodies Prevalence

The best model indicated that four of the five explanatory variables contributed to explaining the variation of EBLV-1 seroprevalence (Table 4). These four variables (taxon, month, colony size and species richness) had high Akaike importance weights (∑w_i_ > 0.92) and were included in all high ranking models. The two best-fitting models (ΔAICc<2) explained >90% of the seroprevalence variability observed and both included these four variables. Inclusion of the variable sex (∑w_i_ = 0.43) produced an equally valid model (χ^2^ = 2.18, df = 1, p = 0.14) but fit the data less well. Although the model with one interaction was a little better, we retained the first model without interaction to explain the EBLV-1–seroprevalence variation because it did not differ significantly from the former and it was more parsimonious.

**Table 4:** Summary of the ten first models and two best-supported models (ΔAICc<2) fitted to estimate variations in EBLV-1 prevalence in bats. In bold the best model selected.

Models	AICc	ΔAICc	w_i_
taxon + colony size + species richness + month	1914.390	0.000	0.533
taxon + sex + colony size + species richness + month	1915.016	0.626	0.390
taxon + colony size + month	1919.562	5.172	0.040
taxon + sex + colony size + month	1919.752	5.362	0.037
taxon + colony size	1931.116	16.726	0.000
taxon + sex + colony size	1931.414	17.024	0.000
taxon + colony size + species richness	1931.581	17.191	0.000
taxon + sex + colony size + species richness	1931.816	17.426	0.000
sex + colony size + species richness + month	1941.733	27.343	0.000
colony size + species richness + month	1944.736	30.346	0.000

The results obtained with the best model indicated that EBLV-1 seroprevalence varied widely among months, with July having highest seroprevalence. Seroprevalence also differed among the taxon, with the *Vespertilionidae* family having the highest EBLV-1 seroprevalence, compared to other families, that was significantly higher than those of *Rhinolophidae* and *Molossidae* families (McNemar test, p<0.001 for both).

**Figure 2:**
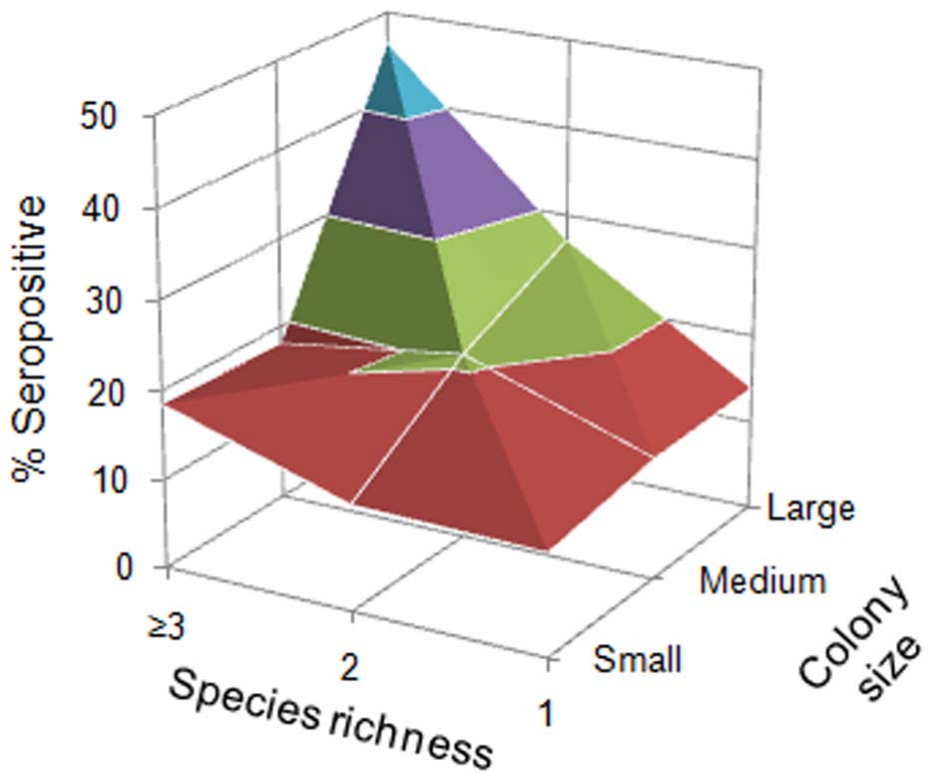
Variations of the percentages of seropositive bats as a function of the species richness and colony size. Seropositive rates are as follows: Red, 10–20%; green, 20–30%, purple, 30–40%; blue, 40–50%.

The estimated β-coefficients indicated that seroprevalence was positively associated with colony size. EBLV-1 seroprevalence was significantly higher in medium and large colonies than small colonies (Figure 2, Table 5). Bats living in medium (ORs =  1.96) or large colonies (ORs =  4.50) had, respectively, nearly two and more than four times greater probability of being seropositive than bats roosted in small colonies. Seroprevalence also increased with the species richness present in the colony but was significantly higher only when the colonies were constituted of three or more species (β = 0.88, p<0.001). In these cases, bats had more twice higher probability of being seropositive (ORs = 2.42) than in monospecific colonies.

**Table 5:** Parameter estimates (logit scale) from the best model on the seroprevalence of EBLV-1.

Explanatory variables	β	error	odds-ratio	95 CI	z-value	*p*
intercept	−4.310	0.667			−6.463	< 0.001
taxon						
*Rhinolophidae* *						
*Vespertilionidae*	0.992	0.307	2.696	1.475–4.927	3.225	0.001
*Miniopteridae*	0.129	0.322	1.138	0.606–2.138	0.403	0.687
*Molossidae*	−0.158	0.394	0.853	0.394–1.847	−0.402	0.687
months						
April*						
May	0.612	0.504	1.844	0.686–4.952	1.215	0.224
June	0.791	0.515	2.205	0.804–6.048	1.537	0.124
July	1.499	0.517	4.480	1.625–12.347	2.899	0.004
August	0.519	0.530	1.681	0.594–4.753	0.980	0.327
September	1.118	0.692	3.059	0.788–11.878	1.615	0.106
October	0.449	0.573	1.567	0.509–4.816	0.784	0.433
species richness						
1 species*						
2 species	0.149	0.227	1.160	0.744–1.809	0.657	0.511
≥ 3 species	0.883	0.234	2.419	1.527–3.831	3.767	< 0.001
colony size						
Small*						
medium	0.674	0.214	1.962	1.289–2.896	3.146	0.002
large	1.506	0.209	4.501	2.994–6.786	7.214	< 0.001
random effects	σ^2^	error				
locality	0.000	0.000				
year	1.344	1.159				

* Reference.

## Discussion

We completed our active surveillance of Spanish bat colonies that began in 1992 [17] and this analysis extends our knowledge of EBLV-1 infection in bats. Herein, we report the detection of specific EBLV-1–neutralizing antibodies in seven bat species and a considerably higher number of species exposed to *Lyssavirus* in Europe than previously described (Table 3). The high percentage (65%) of seropositive species found suggests that most Spanish species of bats can be exposed to EBLV-1 (Table 1). Even EBLV-1 neutralizing antibodies were identified in *M. daubentonii*, a species so far linked only to EBLV-2 infection in more northern parts of Europe [40],[42],[44] (Table 3). Furthermore, evidence of EBLV-1 infection was found in 68% of the bat colonies sampled. These findings are in agreement with a wide geographic distribution of EBLV-1 infection of bats in the Spanish Mediterranean region [17],[35]–[37] (Figure 1).

Our results indicate that EBLV-1–seroprevalence varies among bats at a national scale were associated with several ecological factors operating at species and community levels, including breeding period, taxonomic family, colony size, and species richness in the colony. Our results showed that EBLV-1 seroprevalence varied broadly among the bat species and localities sampled. Previous studies demonstrated that bat *Lyssavirus* dynamics exhibited a strong seasonal pattern [25] and that the breeding period could favor bat infection [26]. This seasonal variability was also detected by our model, which indicated significantly higher EBLV-1 seroprevalence in summer (July), when maternity colonies are present in most of the localities. The model that includes the variable sex was not better than the best model (without sex), suggesting that sex-ratio changes observed during the year did not influence EBLV-1 seroprevalence.

EBLV-1–seroprevalence differences were also found among bat families. This variability might be explained by different susceptibilities to infection or immunological responses of the bat species to EBLV-1 virus. For example, we observed important differences between two species from two families: *R. ferrumequinum* had the highest percentage of positive clots and organs compared to other species, e.g., *M. myotis*, while the percentage of seropositive *R. ferrumequinum* was much lower than that of *M. myotis*. Our results are less in favor of the hypothesis of the difference in susceptibility because *R. ferrumequinum* bats were infected (nRT-PCR–positive in this study). These differences might rather suggest different seroconversion rates in these two species. In this sense, Turmelle et al. [45] reported that significant differences in seroconversion probabilities were found among bats depending on whether they had previously been infected, suggesting that long-term repeated infections of bats might confer significant immunological memory and reduced susceptibility to rabies infection. Immune competence in bats can vary with body condition (via nutritional status and stress) and reproductive activity and, as a consequence, can lead to a lower rabies seroprevalence between or within bat species [26],[46],[47].

Determining whether these differences are a consequence of ecological, immunological or phylogenetic factors is very difficult. Perhaps the phylogenetic distance between the *Rhinolophidae* and *Vespertilionidae* contributes to these differences [27], as was shown in studies on Coronavirus in bats [48]. However, further studies are needed to investigate this hypothesis.

Our analyses revealed that the colony size and species richness it harbored were two important ecological factors and showed their relevant roles in seroprevalence variability. Notably, EBLV-1 seroprevalence and the colony size, especially large colonies, had a strong positive association. Previous studies suggested that larger colony size could also raise host density and simultaneously favor contact rates between individuals and, hence, the probability of infection spread [49]–[51]. However, colony size alone could not explain all the variability observed, especially when the colonies were comprised principally of one species, suggesting that other factors might be involved in bat seroprevalence. In this sense, Streicker et al. [52] showed that rabies virus (RABV) seroprevalence in common vampire bats was independent of bat-colony size. The absence of a relationship between RABV seroprevalence and colony size in that study could be explained by *Desmodus rotundus* generally forming small- or medium-sized monospecific colonies. Our results suggested that EBLV-1 seroprevalence was strongly affected by the colony size and species richness, and indicated that multispecies, large colonies, especially those with three or more different bat species, had a higher probability of EBLV-1 infection (Figure 2). Large colonies and multispecies associations occurred frequently among cave-dwelling bats, principally during the maternity period. This colonial behavior confers thermodynamic and social advantages to reproductive females during pregnancy and lactation [53]. Higher seroprevalence was observed in multispecies colonies compared to monospecific colonies, suggesting that interspecific virus transmission plays an important role in EBLV-1 dynamics. A higher number of species might not only increase the rates of contact between bat groups but could also facilitate virus entry or spread through the higher mobility of individuals among colonies, especially if there are migratory species.

Cross et al [54] showed that the probability of a pandemic event depended on the interaction between colony size and movement of hosts among groups during their infectious lifetime. They suggested that large groups and frequent movements were more heavily impacted by acute diseases than hosts with small groups and infrequent movement. This could explain the high EBLV-1 seroprevalence observed in large multispecific colonies comprising *M. schreibersii* and, sometimes, *M. capaccinii*, both species being considered regionally migratory [15]. Indeed, these species migrate seasonally a few hundred kilometers [55],[56], and even between Balearic Islands [19]. This migratory behavior can be important for EBLV-1 dispersion within colonies or among localities at local and regional scales [17].

The results obtained since 1992 showed that *M. myotis* is an important species for epidemiological studies of lyssaviruses [17],[19],[35]. Its wide geographical distribution in Europe, high percentage (40.2%) of seropositive individuals, long lifespan of *Lyssavirus* neutralizing antibodies [19] and that it almost always forms multispecific colonies, principally with migratory species, make *M. myotis* a good sentinel species (Table 1).

The ability of bats to occupy man-made structures is of particular importance to public health, because it can increase the probability of contact with domestic animals and humans. However, all synanthropic colonies found during our active surveillance were monospecific. *P. pipistrellus*, one of the most abundant species in southern Europe, has strong synanthropic behavior. In accordance with our results, this species might be less exposed to lyssavirus than cave-dwelling bats because it colonizes buildings and its colonies are often monospecific, small, highly philopatric. These observations could be indicative of a low public health risk associated with *P. pipistrellus*.

The integration of wildlife ecology, behavior and disease dynamics is a relatively new area of research. This approach illustrates the pertinent contribution of integrating ecology and epidemiology to enhancing our understanding of complex multi-host epidemiological systems for bat lyssaviruses. The results provide a number of novel insights and improve our knowledge of bat *Lyssavirus* dynamics.

## Supporting Information

Figure S1 NJ phylogenetic tree using 122-nucleotide long sequence obtained from the blood clots.
(DOC)

Table S1 Details of the total 74 sampling events yielding 2144 of sera samples from 2001 to 2010.
(DOC)
