# Supplementary material for: Ecological Factors Associated with European Bat Lyssavirus Seroprevalence in Spanish Bats
Source: PLoS One. 2013 May 20;8(5):e64467. doi: 10.1371/journal.pone.0064467 (PMC3659107; doi:10.1371/journal.pone.0064467)
Supplement: Figure S1 — NJ phylogenetic tree using 122-nucleotide long sequence obtained from the blood clots. (DOC) [file pone.0064467.s001.doc]

Figure S1. NJ phylogenetic tree using 122-nucleotide long sequence obtained from the blood clots^1^.


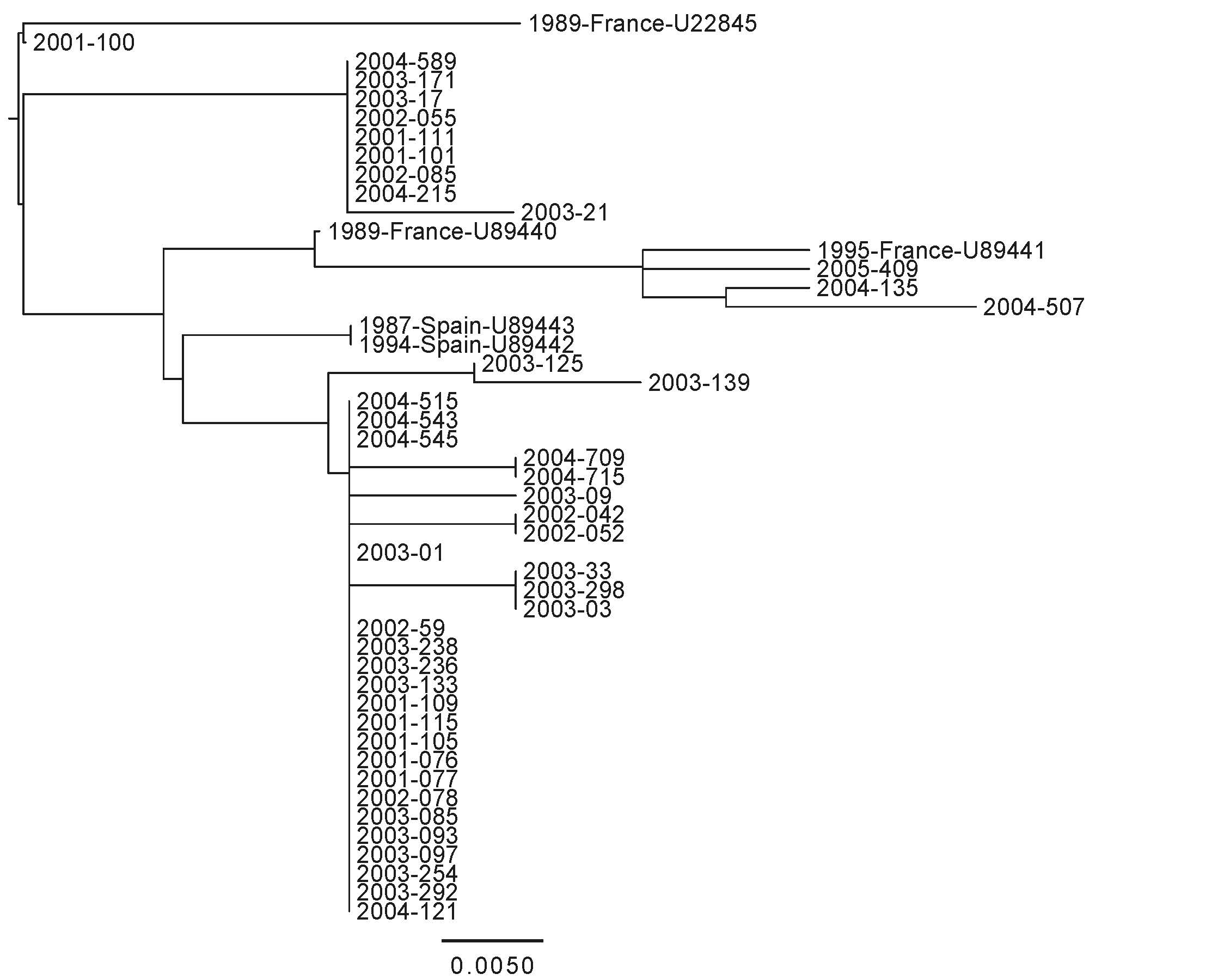


^1^Fifteen sequences have been published in Genbank (EF187833- EF187840, EF187842- EF187847 and EF207412). Unfortunately, to date, Genbank have a policy not to accept sequences shorter than 200 bp. For this reason, the other 28 sequences were not accepted in Genbank.
