# Supplementary material for: Ecological Factors Associated with European Bat Lyssavirus Seroprevalence in Spanish Bats
Source: PLoS One. 2013 May 20;8(5):e64467. doi: 10.1371/journal.pone.0064467 (PMC3659107; doi:10.1371/journal.pone.0064467)
Supplement: Table S1 — Details of the total 74 sampling events yielding 2144 of sera samples from 2001 to 2010. (DOC) [file pone.0064467.s002.doc]

Table S1: Details of the total 74 sampling events yielding 2144 of sera samples from 2001 to 2010.

| **Locality** | **Sampling occasions** | **Years** | **Sr** | **Cs** | **Sampling months** | | | | | | | **N** |
| --- | --- | --- | --- | --- | --- | --- | --- | --- | --- | --- | --- | --- |
|  |  |  |  |  | A | M | J | J | A | S | O |  |
| 1 | 1 | 2002 | 2 | s/l | X |  |  |  |  |  |  | 36 |
| 2 | 4 | 2007-2010 | 1 | s/m |  | X | X_2_ |  |  |  | X | 109 |
| 3 | 3 | 2008-2010 | 2 | l |  |  | X | X | X |  |  | 96 |
| 4 | 2 | 2008, 2010 | 2 | m |  |  | X |  | X |  |  | 49 |
| 5 | 4 | 2007-2010 | 2 / ≥3 | s/m |  |  |  |  |  | X | X_3_ | 118 |
| 6 | 11 | 2001-2010 | 1 | s/m | X_2_ | X |  | X_7_ | X |  |  | 223 |
| 7 | 15 | 2001-2010 | ≥3 | s/m/l | X | X_8_ | X | X_4_ | X |  |  | 614 |
| 8 | 15 | 2001-2010 | 2 | s/m |  | X_3_ | X_2_ | X_7_ | X_3_ |  |  | 273 |
| 9 | 1 | 2010 | 1 | m |  |  | X |  |  |  |  | 20 |
| 10 | 1 | 2002 | 2 | m |  |  |  |  | X |  |  | 35 |
| 11 | 9 | 2004-2010 | 1 | s/l |  | X | X_6_ | X |  |  | X | 408 |
| 12 | 4 | 2006-2009 | 1 | m |  |  | X_3_ | X |  |  |  | 103 |
| 13 | 1 | 2006 | 1 | s |  |  | X |  |  |  |  | 6 |
| 14 | 1 | 2010 | 1 | s |  |  |  |  | X |  |  | 12 |
| 15 | 1 | 2010 | 1 | s |  |  |  |  | X |  |  | 12 |
| 16 | 1 | 2003 | 1 | s |  |  |  |  |  | X |  | 30 |
| **Total** | **74** | **2001-2010** |  |  | **4** | **14** | **18** | **21** | **10** | **2** | **5** | **2.144** |

Sr: category of species richness. Cs: category of colony size (s: small; m: medium; l: large). N: number of bats analyzed. X_n_: indicate the number of sampling occasions for each month.
